# Gait and balance outcomes of group-based music therapy, physiotherapy, and simplified dance training in patients with Parkinson's disease

**DOI:** 10.3389/fresc.2026.1879749

**Published:** 2026-07-16

**Authors:** Kirsti Pedak, Toomas Toomsoo, Indrek Rannama, Maria Ines de Magalhaes Cardoso de Oliveira Margato, Alice Pehk

**Affiliations:** 1 School of Natural and Health Sciences, Tallinn University, Tallinn, Estonia; 2 LTD Confido Medical Centre, Tallinn, Estonia; 3 Music Therapy Centre, Tallinn, Estonia

**Keywords:** balance, gait, music therapy, Parkinson's disease, physiotherapy, rehabilitation, simplified dance training

## Abstract

**Introduction:**

To compare the effects of group-based music therapy, physiotherapy, and simplified dance training on gait, balance, and lower limb force production in individuals with moderate Parkinson's disease.

**Methods:**

Forty-eight participants (Hoehn & Yahr stages 2.0–3.0) were randomly allocated to music therapy (*n* = 14), physiotherapy (*n* = 14), simplified dance training (*n* = 10), or control (*n* = 10). All active groups completed two 60-minute group sessions per week for eight weeks. Outcome measures included static balance assessed via force-plate Centre of Pressure (CoP) metrics, the Five Times Sit-to-Stand test (5STS), and the Timed Up and Go test (TUG) with OptoJump gait analysis. Between-group differences were analyzed using baseline-adjusted analysis of covariance (ANCOVA), with Benjamini–Hochberg false discovery rate correction applied within each outcome family.

**Results:**

Significant baseline-adjusted between-group effects were observed. Physiotherapy showed a lower adjusted center-of-pressure sway area than control (53% lower, *q* = 0.027) and lower weight-distribution asymmetry (62% lower, *q* = 0.050). Both music therapy and simplified dance training had shorter adjusted five-times sit-to-stand times than control (25% and 26% shorter, *q* = 0.036), with no between-group differences in peak force or rate of force development. All three interventions had shorter adjusted Timed Up and Go times than control (*q* = 0.005), the largest difference in simplified dance training (−2.5 s); simplified dance training alone had a shorter adjusted turning time than control (*q* = 0.047), and music therapy alone had a lower adjusted double support time (*q* = 0.030).

**Discussion:**

Each modality produced a distinct, modality-specific pattern of benefit. Physiotherapy improved static postural control and weight-bearing symmetry; both music therapy and simplified dance training improved sit-to-stand performance; and all three improved overall mobility, with the greatest gains in dance training for gait and turning. These findings support a personalized, potentially multimodal approach to Parkinson's disease rehabilitation. The study was registered retrospectively in the World Health Organization International Clinical Trials Registry Platform (Registration No. DRKS00039213). All participants provided written informed consent.

## 1 Introduction

Parkinson's disease (PD) is a progressive neurodegenerative disorder. Its global prevalence has increased 2.5-fold over the past three decades, and it now affects more than six million individuals worldwide (1). PD is characterized by the cardinal motor features of bradykinesia, rigidity, resting tremor, and postural instability. Together, these features contribute to impaired postural control and balance (2, 3). Such deficits significantly increase fall risk and reduce independence. This makes the assessment and management of axial motor symptoms a key clinical priority (4–6).

A major challenge in PD management is the limited effectiveness of dopaminergic pharmacotherapy on axial symptoms such as gait and postural control. Levodopa effectively improves appendicular motor function. However, its impact on balance and gait is often inconsistent and may be complicated by motor fluctuations and dyskinesia (7, 8). Consequently, there is growing emphasis on non-pharmacological interventions. Physical exercise is now recognized as a central component of PD rehabilitation. Evidence suggests that it promotes neuroplasticity through mechanisms such as synaptogenesis, neurogenesis, and strengthening of motor circuitry (9–11). These adaptations may support cortical compensation and improve the automaticity of movement.

Meta-analyses have demonstrated that balance- and gait-oriented exercise interventions significantly improve postural control and functional mobility in individuals with PD (12, 13). Group-based rehabilitation approaches may offer additional advantages. By combining structured training with social interaction and peer support, they can also enhance adherence (14). Beyond conventional physiotherapy, there is increasing interest in performing arts–based interventions, such as dance and music therapy. These approaches engage motor, cognitive, emotional, and social domains simultaneously (15).

Dance-based interventions incorporate rhythmic cueing, coordination, and multidirectional movement. These elements lead to improvements in balance, motor function, and cognitive performance (16–18). Long-term participation has also been associated with neuroplastic changes in motor and multisensory integration networks, as well as enhanced functional mobility and quality of life (19). Music-based interventions, particularly Neurologic Music Therapy (NMT) and Rhythmic Auditory Stimulation (RAS), use auditory–motor entrainment to improve the temporal aspects of gait. They provide external timing cues that compensate for impaired basal ganglia function (20, 21). Recent systematic reviews and meta-analyses further demonstrate that music-based interventions significantly improve gait velocity, stride length, and balance performance in people with PD (22, 23).

Despite this growing body of evidence, the relative efficacy of these approaches compared with conventional physiotherapy remains uncertain. Few studies have directly compared them within the same clinical context. This gap is particularly relevant for group-based interventions, where social and motivational factors may further influence outcomes. Therefore, this study compares the effects of an eight-week group-based program of music therapy, structured physiotherapy, and simplified dance training on gait performance, balance, and lower limb function in individuals with moderate-stage PD. An inactive control group served as the comparison condition.

## 2 Methods

### 2.1 Participants

The study recruited individuals diagnosed with Parkinson's disease classified as Hoehn and Yahr (HY) stages 2.0–3.0 (24) from the database of AS Confido Medical Centre. A neurologist assessed eligibility and invited suitable candidates. Inclusion required a clinically confirmed PD diagnosis based on Movement Disorder Society criteria and the capacity to provide written informed consent.

All participants continued their prior medication regimens under neurological supervision; none had prior exposure to the intervention types used. All assessments were performed during the medication “ON” state at both baseline and post-intervention. Baseline characteristics are presented in Table 1.

**Table 1:** Baseline characteristics of participants by intervention group.

Variables	Music Therapy (M ± SD)	Physiotherapy (M ± SD)	Dance Therapy (M ± SD)	Control Group (M ± SD)
n	14	14	10	10
Weight (kg)	75.93 (15.11)	77.50 (13.58)	72.30 (14.02)	77.90 (16.14)
Height (cm)	171.14 (10.78)	170.50 (11.18)	173.20 (8.84)	171.20 (7.87)
Age at inclusion (years)	67.42 (11.61)	68.16 (9.86)	69.20 (11.76)	70.30 (7.86)
Age at diagnosis (years)	62.00 (12.87)	63.93 (8.92)	64.40 (11.46)	66.70 (7.26)
Disease duration (years)	5.43 (2.65)	3.86 (2.41)	4.80 (3.23)	4.60 (1.83)
Hoehn & Yahr	2.79 (0.43)	2.43 (0.51)	2.40 (0.52)	2.40 (0.52)
Levodopa dose (mg/day)	475 (180.54)	367.86 (144.92)	448 (187.78)	360 (157.76)

M, Mean; SD, Standard Deviation; HY, Hoehn & Yahr scale.

The study protocol was approved by the Human Research Ethics Committee of the Estonian National Institute for Health Development (Approval No. 1100) and conducted in accordance with the Declaration of Helsinki. The study was retrospectively registered in the World Health Organization International Clinical Trials Registry Platform (Registration No. DRKS00039213). All participants provided written informed consent.

### 2.2 Randomization and blinding

The allocation sequence was generated in Microsoft Excel by a data analyst external to the research team who was not involved in participant recruitment, intervention delivery, or outcome assessment, thereby ensuring allocation concealment from the enrolling staff. Eligible participants were randomly allocated to one of four groups: music therapy, physiotherapy, simplified dance training, or an inactive control group.

**Figure 1:**
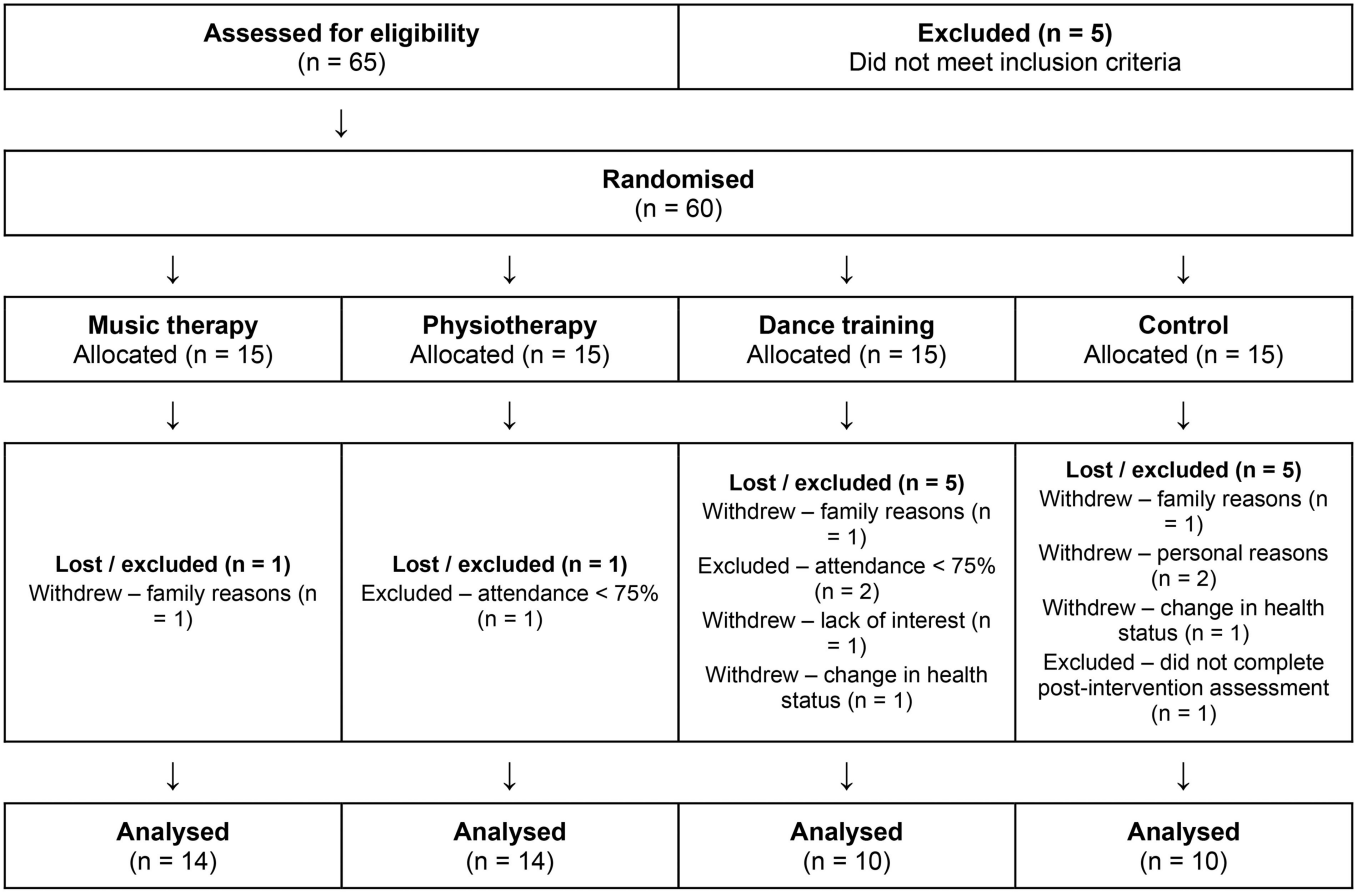
CONSORT participant flow diagram.

Of the 65 individuals assessed for eligibility, five did not meet the inclusion criteria. The remaining 60 participants were randomized in equal numbers (*n* = 15 per group). After randomization, 12 participants were lost or excluded: three for family reasons, three for attending fewer than 75% of the planned sessions, two for a change in health status, two for personal reasons, one lacking interest, and one for not completing the post-intervention assessment. The final analytic sample comprised 48 patients (21 men, 27 women): 14 in music therapy, 14 in physiotherapy, 10 in simplified dance training, and 10 in the control group. The participant flow is presented in Figure 1.

Participants were unaware of their group assignment until the baseline assessments had been completed. The assessor who administered and analyzed the force-plate and OptoJump measures remained blinded to group allocation throughout both the baseline and post-intervention assessments.

### 2.3 Assessment protocol

Physical and motor performance was evaluated before and after the intervention using three standardized tests administered in fixed order: the Static Balance Test (SB), the Five Times Sit-to-Stand test (5STS), and the Timed Up and Go test (TUG). Each test was preceded by a familiarization phase detailing procedures and equipment.

### 2.4 Outcome measures

#### 2.4.1 Static balance test (SB)

Participants stood on two eight-channel Kistler force plates (Type 9286A; Kistler Instrument AG, Switzerland) with feet parallel (−20 cm apart) and arms relaxed at the sides for 20 s as motionless as possible. Four trials were performed with 3 min rest between trials. Ground reaction force (GRF) signals were acquired at 200 Hz via a Kistler 64-channel data acquisition (DAQ) system and exported in C3D format for processing with Visual3D Professional V6 (C-Motion Inc.). All signals were filtered with a 10 Hz fourth-order Butterworth low-pass filter. The best trial—defined by the smallest CoP 95% confidence ellipse area—was selected for analysis. Outcomes included: (i) CoP area (mm^2^), the area of the 95% confidence ellipse of the CoP trajectory; (ii) vCoP (mm/s), mean velocity of CoP displacement; and (iii) ASIWD (%, absolute symmetry index of weight distribution between left and right lower limbs.

#### 2.4.2 Five times sit-to-stand test (5STS)

Participants performed five consecutive sit-to-stand movements as rapidly as possible from a standardized 46 cm armless chair positioned behind the force plates. An LED light, synchronized with the Kistler DAQ system, at 1.2 m height and 1.5 m distance provided a visual start signal concurrent with a verbal command (25). Two test trials were recorded after one familiarization trial, with 3 min rest between attempts. GRF signals were processed identically to the SB protocol. Custom pattern-recognition algorithms identified movement phases from a 2 Hz fourth-order Butterworth-filtered, body-weight-normalized vertical GRF signal. Outcomes included: (i) ttot-5STS (s), total test completion time; (ii) Fmax (BW), mean maximal vertical force across five repetitions; and (iii) RFD (BW/s), average rate of force development during the concentric phase.

#### 2.4.3 Timed Up and Go test (TUG)

The TUG was used to assess functional mobility (26) and step characteristics. A 3 m walkway was demarcated with a cone in front of an armless chair. Participants stood, walked to and around the cone, returned, and sat down at a comfortable self-selected pace. Timing ran from movement initiation to first seat contact. Performance was captured at 60 Hz by a webcam positioned 4.5 m from the turn point. Turning time (tturn-TUG) was extracted using Kinovea 0.9.5 freeware. Step characteristics during TUG were recorded with an OptoJump® system (Microgate, Bolzano, Italy), providing gait cycle duration (GCD (s)), double support time (DST (%GC)), and stance phase duration (SPD (%GC)). Two test trials following two familiarizations were averaged.

### 2.5 Intervention programs

After the baseline assessment, participants proceeded to their allocated intervention group. All active interventions consisted of two 60 min group sessions per week for eight weeks and were delivered by certified professionals with extensive clinical experience in their respective fields. The physiotherapist held a relevant degree and a postgraduate qualification in neurological rehabilitation, with nearly 20 years of clinical experience in Parkinson's disease rehabilitation. The music therapist held a doctoral degree in music therapy and a Level 7 certification, with over 30 years of clinical practice. The dance training was led by a professional choreographer and folk dance educator with more than a decade of academic teaching experience and over 30 years of practice, with recognized expertise in folk dance. Each intervention was delivered by the same professional throughout the entire eight-week period, ensuring consistent session structure, exercise progression, and participant guidance. Control participants maintained their usual daily routines with no supplementary intervention.

#### 2.5.1 Music therapy

Sessions were grounded in standardized NMT protocols (27) and receptive relaxation techniques (28). Each session began with a brief warm-up to establish a safe group atmosphere and a participant self-assessment to tailor activities to current needs. The active phase incorporated Therapeutic Instrumental Music Performance (TIMP) to enhance motor control and Patterned Sensory Enhancement (PSE) to guide movement via rhythmic and melodic cues. Vocal exercises included Therapeutic Singing (TS) and Vocal Intonation Therapy (VIT) to support phonation and breathing function. Cognitive stimulation was provided through Musical Attention Control Training (MACT) and Musical Executive Function Training (MEFT). Sessions concluded with receptive relaxation and a debriefing phase.

#### 2.5.2 Physiotherapy

Physiotherapy sessions focused on balance, coordination, and breathing, with exercise selection guided by published balance and gait rehabilitation literature (30–32). Each 60 min session comprised a warm-up (5 min), main training (50 min), and cool-down (5 min). The main phase included general balance and coordination exercises (10 repetitions each, performed at a comfortable pace and then at increased tempo), vestibular activation (head movements with eyes open and closed, difficulty adapted to individual capacity), and movement accuracy and rhythmic control drills. Sessions ended with breathing exercises combined with low-intensity whole-body movements.

#### 2.5.3 Simplified dance training

The simplified dance training was based on movements derived from traditional Estonian folk and social dances, selected for their cultural familiarity and low-performance-pressure context. Each person with PD was paired with the same healthy volunteer partner throughout the eight weeks. Sessions incorporated multidirectional movements—turning, stepping in columns, circles, and formations—to live music accompaniment. Partner-based formations (contra dances) and step sequences integrated walking, dance-specific footwork, and rhythmic alternation. Primary training goals were rhythmic and tempo adaptation, step-sequence memorization, spatial awareness, and partner coordination, consistent with evidence-based approaches in the dance-based intervention literature.

### 2.6 Statistical analysis

Statistical analyses were performed in JASP (version 0.96). Between-group effects were analyzed using baseline-adjusted ANCOVA models. For each outcome, the post-intervention value was entered as the dependent variable, the corresponding baseline value as a covariate, and group as the fixed factor. The main effect of interest was the baseline-adjusted group effect. Where this effect was significant, pairwise comparisons between each active intervention and control were examined using mean differences (MD) between adjusted marginal means with Holm correction.

Right-skewed outcomes were transformed using the natural logarithm before inferential modeling. Log transformation was applied to CoP area, vCoP, ASIWD, and 5STS total time. For these outcomes, both baseline and post-intervention values were log-transformed, and inferential conclusions were based on the transformed ANCOVA models. Adjusted marginal means are reported in the original measurement units after back-transformation. Model assumptions were assessed using residual diagnostics, influential observations, and Levene's test for homogeneity of variance.

Benjamini-Hochberg false discovery rate (FDR) correction was applied within each predefined outcome family: static balance, 5STS performance, and TUG/gait parameters. Correction was applied to the raw *p*-values of the omnibus group effect from the ANCOVA models. Tables report raw *p*-values, FDR-corrected q-values, and partial eta squared (*η*p^2^). Statistical significance was interpreted using *q* < 0.05.

No formal *a priori* sample-size calculation was performed because recruitment was constrained by the available local Estonian Parkinson's disease population. The final sample represented approximately 10% of the local diagnosed Parkinson's disease population; therefore, the study was considered exploratory and hypothesis-generating. *post-hoc* power based on observed effects was not calculated.

## 3 Results

### 3.1 Static balance

Baseline-adjusted static balance outcomes are presented in Table 2. A significant group effect was observed for log-transformed CoP area, F(3,43) = 4.352, *p* = 0.009, *q* = 0.027, *η*p^2^ = 0.233. Pairwise comparisons showed that physiotherapy had a significantly lower baseline-adjusted CoP area than control, ln MD = −0.761, SE = 0.211, t(43) = −3.612, Holm-adjusted *p* = 0.005, corresponding to an estimated 53.3% lower CoP area relative to control. Music therapy and simplified dance training did not differ significantly from control after Holm correction, *p* = 0.251 and *p* = 0.286, respectively.

**Table 2:** Baseline-adjusted ANCOVA results for static balance outcomes.

Parameter	Music therapy	Physio-therapy	Simplified dance training	Control	F(3,43)	*p*	q	*η*p^2^	Levene p
CoP area (mm^2^) #	138.8 (105.5, 182.6)	99.4 (75.4, 131.4)	137.5 (98.3, 192.5)	212.9 (153.6, 295.3)	4.352	0.009	0.027	0.233	0.868
vCoP (mm/s) #	13.4 (11.4, 15.8)	15.0 (12.6, 17.7)	17.2 (14.2, 20.8)	15.7 (12.9, 19.1)	1.425	0.249	0.249	0.09	0.82
ASIWD (%) #	9.87 (6.58, 14.78)	5.98 (3.99, 8.96)	9.50 (5.89, 15.30)	15.63 (9.64, 25.33)	3.178	0.033	0.05	0.181	0.014

Values are baseline-adjusted post-intervention estimated marginal means with 95% CI. For ln-transformed ANCOVA models (#), adjusted means and CIs are back-transformed and should be interpreted as geometric adjusted means. q-values are Benjamini–Hochberg FDR-adjusted within the outcome family. Levene *p* refers to the equality-of-variances check for the model.

A significant group effect was also found for log-transformed ASIWD, F(3,43) = 3.178, *p* = 0.033, *q* = 0.050, *η*p^2^ = 0.181. Pairwise comparisons indicated that physiotherapy had significantly lower baseline-adjusted ASIWD than control, ln MD = −0.960, SE = 0.315, t(43) = −3.053, Holm-adjusted *p* = 0.023, corresponding to an estimated 61.7% lower ASIWD relative to control. Music therapy and simplified dance training did not differ significantly from control, both Holm-adjusted *p* = 0.575. However, because Levene's test indicated heterogeneity of variance for ASIWD, *p* = 0.014, this result should be interpreted cautiously.

No significant group effect was observed for log-transformed vCoP, F(3,43) = 1.425, *p* = 0.249, *q* = 0.249, *η*p^2^ = 0.090.

### 3.2 Five times sit-to-stand

Table 3 presents baseline-adjusted 5STS outcomes. A significant group effect was observed for log-transformed 5STS total time, F(3,43) = 4.077, *p* = 0.012, *q* = 0.036, *η*p^2^ = 0.221. Pairwise comparisons showed that both music therapy and simplified dance training had significantly shorter baseline-adjusted 5STS total times than control. For music therapy, ln MD = −0.293, SE = 0.093, t(43) = −3.145, Holm-adjusted *p* = 0.018, corresponding to an estimated 25.4% shorter 5STS time than control. For simplified dance training, ln MD = −0.300, SE = 0.100, t(43) = −2.984, Holm-adjusted *p* = 0.023, corresponding to an estimated 25.9% shorter 5STS time than control. Physiotherapy did not differ significantly from control after Holm correction, ln MD = −0.215, SE = 0.093, t(43) = −2.310, Holm-adjusted *p* = 0.103.

**Table 3:** Baseline-adjusted ANCOVA results for five times Sit-to-stand outcomes.

Parameter	Music therapy	Physio-therapy	Simplified dance training	Control	F(3,43)	*p*	q	ηp^2^	Levene p
5STS total time (s) #	9.88 (8.76, 11.16)	10.68 (9.46, 12.06)	9.82 (8.50, 11.32)	13.24 (11.47, 15.29)	4.077	0.012	0.036	0.221	0.762
Fmax (BW)	1.24 (1.22, 1.26)	1.22 (1.20, 1.24)	1.22 (1.19, 1.24)	1.23 (1.21, 1.25)	1.099	0.36	0.36	0.071	0.324
RFD (BW/s)	6.75 (6.16, 7.33)	6.65 (6.07, 7.24)	6.97 (6.28, 7.67)	7.61 (6.91, 8.31)	1.697	0.182	0.273	0.106	0.28

Values are baseline-adjusted post-intervention estimated marginal means with 95% CI. For ln-transformed ANCOVA models (#), adjusted means and CIs are back-transformed and should be interpreted as geometric adjusted means. q-values are Benjamini–Hochberg FDR-adjusted within the outcome family. Levene *p* refers to the equality-of-variances check for the model.

No significant group effects were observed for Fmax, F(3,43) = 1.099, *p* = 0.360, *q* = 0.360, *η*p^2^ = 0.071, or RFD, F(3,43) = 1.697, *p* = 0.182, *q* = 0.273, *η*p^2^ = 0.106.

### 3.3 Timed Up and Go and gait parameters

Baseline-adjusted TUG and gait outcomes are shown in Table 4. A significant group effect was observed for total TUG time, F(3,43) = 9.568, *p* < 0.001, *q* = 0.005, *η*p^2^ = 0.400. Pairwise comparisons showed that all three active interventions had significantly shorter baseline-adjusted TUG times than control. Music therapy differed from control by MD = −1.677 s, SE = 0.455, t(43) = −3.684, Holm-adjusted *p* = 0.003. Physiotherapy differed from control by MD = −1.771 s, SE = 0.453, t(43) = −3.908, Holm-adjusted *p* = 0.002. Simplified dance training showed the largest adjusted difference relative to control, MD = −2.524 s, SE = 0.490, t(43) = −5.151, Holm-adjusted *p* < 0.001.

**Table 4:** Baseline-adjusted ANCOVA results for Timed Up and Go and step (gait) parameters.

Parameter	Music therapy	Physio-therapy	Simplified dance training	Control	F(3,43)	*p*	q	*η*p^2^	Levene p
TUG total time (s)	9.24 (8.64, 9.83)	9.14 (8.55, 9.73)	8.39 (7.69, 9.09)	10.91 (10.22, 11.61)	9.568	<0.001	0.005	0.4	0.665
TUG turning time (s)	1.32 (1.18, 1.47)	1.33 (1.19, 1.47)	1.22 (1.05, 1.38)	1.57 (1.41, 1.74)	3.332	0.028	0.047	0.189	0.005
Gait cycle duration (s)	0.95 (0.91, 1.00)	0.96 (0.92, 1.01)	0.95 (0.90, 1.00)	0.98 (0.93, 1.03)	0.287	0.834	0.834	0.02	0.245
Double support time (%GC)	27.6 (25.9, 29.2)	28.4 (26.7, 30.0)	28.4 (26.3, 30.5)	31.7 (29.8, 33.6)	4.096	0.012	0.03	0.222	0.059
Stance phase duration (%GC)	64.8 (63.8, 65.8)	64.2 (63.1, 65.2)	65.5 (64.3, 66.8)	66.1 (65.0, 67.3)	2.546	0.068	0.085	0.151	0.35

Values are baseline-adjusted post-intervention estimated marginal means with 95% CI. q-values are Benjamini–Hochberg FDR-adjusted within the outcome family. Levene *p* refers to the equality-of-variances check for the model.

A significant group effect was also found for TUG turning time, F(3,43) = 3.332, *p* = 0.028, *q* = 0.047, *η*p^2^ = 0.189. Pairwise comparisons showed that simplified dance training had a significantly shorter baseline-adjusted turning time than control, MD = −0.356 s, SE = 0.117, t(43) = −3.037, Holm-adjusted *p* = 0.024. Music therapy and physiotherapy did not differ significantly from control after Holm correction, both *p* = 0.143. Because Levene's test indicated heterogeneity of variance for TUG turning time, *p* = 0.005, this result should be interpreted cautiously.

A significant group effect was observed for double support time, F(3,43) = 4.096, *p* = 0.012, *q* = 0.030, *η*p^2^ = 0.222. Pairwise comparisons showed that music therapy had significantly lower baseline-adjusted double support time than control, MD = −4.169% gait cycle, SE = 1.247, t(43) = −3.343, Holm-adjusted *p* = 0.010. Physiotherapy showed a borderline difference relative to control, MD = −3.341% gait cycle, SE = 1.251, t(43) = −2.670, Holm-adjusted *p* = 0.053, whereas simplified dance training did not differ significantly from control after Holm correction, MD = −3.289% gait cycle, SE = 1.416, t(43) = −2.323, Holm-adjusted *p* = 0.100.

No significant group effects were observed for gait cycle duration, F(3,43) = 0.287, *p* = 0.834, *q* = 0.834, *η*p^2^ = 0.020, or stance phase duration, F(3,43) = 2.546, *p* = 0.068, *q* = 0.085, *η*p^2^ = 0.151.

## 4 Discussion

The principal finding of this study is that music therapy, physiotherapy, and simplified dance training each produced distinct, modality-specific motor benefits relative to control in individuals with moderate Parkinson's disease over the eight-week intervention.

After baseline adjustment and FDR correction, significant group effects were observed for CoP area, ASIWD, 5STS total time, TUG total time, TUG turning time, and double support time. The most consistent baseline-adjusted differences relative to control were observed for physiotherapy in static balance-related outcomes, for music therapy and simplified dance training in 5STS total time, and for all active interventions in total TUG time, with the largest adjusted difference observed in the simplified dance training group. Fmax, RFD, vCoP, gait cycle duration, and stance phase duration did not show significant FDR-corrected group effects. This domain-dependent pattern is consistent with evidence that different exercise modalities preferentially engage different motor and neural processes according to their task demands (17, 34).

### 4.1 Static balance

Improvements in **force-plate measures of static balance** were confined to the physiotherapy group, which showed a lower baseline-adjusted center-of-pressure sway area and lower weight-bearing asymmetry than control, whereas neither music therapy nor simplified dance training differed from control on these measures.

This selectivity matches the content of the physiotherapy program, which emphasized repeated postural adjustment, controlled weight shifting, and alignment work, the demands most directly indexed by force-plate measures of static balance, and is maintaining evidence that balance-specific and aerobic–strength physiotherapy produce the largest reductions in postural sway in Parkinson's disease (12, 32, 33). Although network meta-analyses often rank dance highly on clinical balance scales (29), the divergence here suggests that force-plate measures of quiet-stance sway and inter-limb loading capture a narrower, impairment-level construct that responds preferentially to explicit postural training rather than to rhythmic or choreographic activity (36). The weight-distribution finding should nonetheless be interpreted with caution, as the equality-of-variances assumption was not met for this outcome.

### 4.2 Functional lower-limb performance

Both music therapy and simplified dance training had shorter baseline-adjusted five-times sit-to-stand times than control, whereas physiotherapy did not. Importantly, these gains occurred without accompanying changes in maximal force or rate of force development in any group. This dissociation indicates that faster sit-to-stand performance was driven by improved temporal organization, movement initiation, and coordination rather than by increased muscular strength or explosive force (25). Such a mechanism is consistent with the rhythmic-auditory basis shared by both interventions: externally or musically paced movement supports timely movement initiation and more efficient motor sequencing in Parkinson's disease (19, 22), and dance delivers comparable temporal structuring through music-paced postural transitions.

### 4.3 Mobility, turning, and gait

The clearest cross-modality benefit was for overall mobility: all three active interventions had shorter baseline-adjusted Timed Up and Go times than the control, with the largest adjusted difference in simplified dance training. These baseline-adjusted differences in Timed Up and Go time may be clinically relevant in this population. However, they should be interpreted against published thresholds for meaningful change. As a composite of transfer, straight-line gait, and turning, the Timed Up and Go test can capture gains arising through several mechanisms, which may explain its broad responsiveness (38). The modality-specific structure re-emerged in the gait subcomponents. Among the active interventions, only simplified dance training shortened turning time relative to control. This fits the multidirectional stepping, turning movements, and continuous weight shifting that characterize dance and resemble the demands of turning (17, 37). However, this result should be interpreted cautiously because of the heterogeneity of variance. Conversely, the clearest reduction in double support time was seen with music therapy, the only group to differ from control on this measure. The physiotherapy group showed a borderline reduction and simplified dance training, a smaller change that did not separate from control. This pattern is consistent with improved gait timing and rhythmicity under auditory–motor entrainment (19, 22).

These modality-specific effects are likely supported by different mechanisms of neuroplasticity. Rhythmic auditory interventions—music therapy and the music-paced component of dance—are thought to engage cerebello-thalamo-cortical circuitry, which is relatively spared in Parkinson's disease. This external rhythm gives movement a timing cue that the impaired basal ganglia can no longer provide, helping to restore movement initiation and timing (19, 22, 27).

Partner-based dance also combines proprioceptive, vestibular, visual, and auditory information during constantly changing movements. This multisensory demand is thought to strengthen sensorimotor integration and cortical compensation, which translates most directly into better dynamic mobility and turning (18, 30, 35). The two rhythmic interventions differed in their temporal structure, which may explain their different effects. The steady, metronome-like pacing of music therapy suits the timing demands of straight-line walking and double support. The variable, multidirectional structure of dance, by contrast, matches the turning and reorientation demands of turning (17, 19, 37). Structured physiotherapy, by contrast, focuses on repetitive, stability-oriented postural control. This kind of training promotes use-dependent plasticity in postural and sensorimotor networks, which is best reflected in static balance measures (9, 10). The better postural stability and weight transfer from this training may also help the sit-to-stand and turning parts of the Timed Up and Go test. This could explain why a mainly stability-focused program also improved overall mobility. These divergent mechanisms offer a coherent account of why each modality improved the domain most closely matched to its training content.

### 4.4 Task specificity

Across all domains, each intervention worked best for the outcome that most closely matched its movements. This follows the principle of task specificity in motor rehabilitation. The finding does not overturn the broader view that exercise benefits Parkinson's disease, but refines it: the benefit is real, yet depends on the domain. Treatment choice can therefore be matched to a patient's main deficit.

### 4.5 Control group and baseline level

The improvements over eight weeks may partly reflect the cohort's relatively low baseline function, which left more room for measurable change. The active groups showed consistently better baseline-adjusted outcomes than control on the significant measures, but this contrast should be interpreted cautiously. The control group did not decline uniformly; on some measures, its performance was stable or slightly improved over the assessment period. Repeating the same performance tests can produce learning or practice effects, and day-to-day variation in the medication “ON” state adds further variability. The between-group differences are therefore unlikely to reflect intervention effects alone.

### 4.6 Inter-individual variability

Variability between participants was marked, maintaining the recognized heterogeneity of Parkinson's disease phenotypes, symptom severity, and medication response (39). Recent network meta-analyses similarly conclude that no single modality is universally superior (29, 34). Together, these observations support a personalized or combined approach, in which interventions are matched or sequenced according to each patient's dominant impairment—whether static instability, impaired functional transfer, or difficulty with gait and turning.

### 4.7 Limitations

Several limitations should be noted. First, the group sizes were small, particularly in the simplified dance training and control groups (*n* = 10 each), and no *a priori* power calculation was performed; although the sample represents approximately 10% of the Estonian population diagnosed with Parkinson's disease, the statistical power to detect subtle between-group differences was limited, and some effect estimates should therefore be interpreted with caution. Second, outcomes were assessed only immediately after the eight-week block, with no longer-term follow-up, so the durability of the observed improvements remains unknown, and the intervention period may have been too short to capture the full extent of neuroplastic adaptation. Third, the use of an inactive rather than an active control group limits causal inference, and the single-center design with a relatively homogeneous sample constrains external validity. Finally, home-based physical activity was not continuously monitored in any group during the eight weeks—including the control group, whose members continued their usual daily activities—and individual training workload and intensity were not objectively quantified. In addition, because each intervention was delivered by a single experienced professional, the effect of each modality cannot be fully separated from the influence of the individual instructor, and natural day-to-day fluctuation within the medication “ON” state may have introduced further variability into the performance measures. Future work should employ adequately powered, multi-center designs with longer follow-up and standardized monitoring of activity and workload to determine which patient profiles derive the greatest benefit from specific therapeutic approaches.

## 5 Conclusion

This study indicated that group-based music therapy, physiotherapy, and simplified dance training produce distinct, modality-specific motor benefits in individuals with moderate Parkinson's disease. Physiotherapy improved static postural stability and bilateral weight-bearing symmetry; both music therapy and simplified dance training enhanced functional lower-limb performance; and all three improved overall mobility, with simplified dance training showing the largest gains in gait and turning. Rather than identifying a single superior intervention, the results suggest that different modalities engage complementary motor processes, and that rehabilitation may be most effective when matched to a patient's specific deficits.

Future research should examine whether different Parkinson's disease phenotypes respond differently to music-, dance-, and physiotherapy-based interventions and whether combining these modalities offers added benefit. Studies that include neurophysiological measures are also needed to clarify the mechanisms behind the modality-specific effects.

## Data Availability

The datasets generated and analyzed during the current study are not publicly available because they contain sensitive clinical data from individuals with Parkinson's disease. In accordance with the approval of the Human Research Ethics Committee of the Estonian National Institute for Health Development (Approval No. 1100) and applicable local data-protection regulations, the data are available from the corresponding author on reasonable request and subject to ethics approval.
